# FACT or PACT: A Comparison between Free-Acrylamide and Acrylamide-Based Passive Sodium Dodecyl Sulfate Tissue Clearing for whole Tissue Imaging

**DOI:** 10.22074/cellj.2019.5989

**Published:** 2019-02-25

**Authors:** Huimei Wang, Arezoo Khoradmehr, Amin Tamadon

**Affiliations:** 1 Department of Integrative Medicine and Neurobiology, School of Basic Medical Sciences, Institute of Acupuncture and Moxibustion, Fudan Institutes of Integrative Medicine, Fudan University, Shanghai, China; 2 Research and Clinical Center for Infertility, Shahid Sadoughi University of Medical Sciences, Yazd, Iran; 3 The Persian Gulf Marine Biotechnology Research Center, The Persian Gulf Biomedical Sciences Research Institute, Bushehr University of Medical Sciences, Bushehr, Iran

**Keywords:** Acrylamide, Imaging, Staining and Labeling, Three-Dimensional, Tissues

## Abstract

Major biological processes rely on the spatial organization of cells in complex, highly orchestrated three-dimensional (3D) tissues. Until the recent decade, most of information on spatial neural representation primarily came from microscopic imaging of “2D” (5-50 μm) tissue using traditional immunohistochemical techniques. However, serially sectioned and imaged tissue sections for tissue visualization can lead to unique non-linear deformations, which dramatically hinders scientists’ insight into the structural organization of intact organs. An emerging technique known as CLARITY renders large-scale biological tissues transparent for 3D phenotype mapping and thereby, greatly facilitates structure-function relationships analyses. Since then, numerous modifications and improvements have been reported to push the boundaries of knowledge on tissue clearing techniques in research on assembled biological systems. This review aims to outline our current knowledge on next-generation protocols of fast free-of-acrylamide clearing tissue (FACT) and passive CLARITY (PACT). The most important question is what method we should select for tissue clearing, FACT or PACT. This review also highlights how FACT differs from PACT on spanning multiple dimensions of the workflow. We systematically compared a number of factors including hydrogel formation, clearing solution, and clearing temperatures between free-acrylamide and acrylamide-based passive sodium dodecyl sulfate (SDS) tissue clearing and discussed negative effects of polyacrylamide on clearing, staining, and imaging in detail. Such information may help to gain a perspective for interrogating neural circuits spatial interactions between molecules and cells and provide guidance for developing novel tissue clearing strategies to probe deeply into intact organ.

## Introduction

Biological systems are capable of forming complex neuronal network of feedforward, feedback and horizontal circuits (1), the most prominent biological systems being the neural basis of spatial codes. Decades of research on the neurobiology of tissue imaging mainly focused on neuroanatomical structures by mechanical slicing procedures, which laid the foundations for understanding neural maps of 2D spatial representations. However, tissue opacity and light scattering greatly limit the tissue depth which can be sectioned optically. Furthermore, high- resolution reconstruction techniques for 3D image require sophisticated image computation, which are markedly labor-intensive and time-consuming. Moreover, although several novel brain imaging techniques including magnetic resonance imaging (MRI) (2), computed tomography (CT) (3, 4), positron emission tomography (PET) (5), confocal microscopy (6) and two-photon microscopy (7) have garnered considerable success owing to their higher attainable resolution and deeper penetration depths, high cytoarchitectural resolution and large-volume organ details still remain to be achieved.

An emerging theme is that many optical clearing techniques have been developed recently and refined continually. Of these approaches, benzyl alcohol and benzyl benzoate (BABB) are among the first to make fixed tissues as large as 2 cm^2^ transparent for deep microscopic imaging (8), compared with 5-20 µm sections for conventional immunohistochemical techniques. The potential to analyze complex neural networks make tissue-clearing methods extremely intriguing for subcellular and cellular analyses of complex structures. Enormous advances have since been made for high-resolution and large-scale imaging of tissue clearing, including scale (9), dibenzyl ether (DBE) (10), 3D imaging of solvent-cleared organs (3DISCO) (11, 12), see deep brain (seeDB) (13), ClearT (14), clear unobstructed brain imaging cocktails (CUBIC) (15, 16), system-wide control of interaction time and kinetics of chemical (SWITCH) (17), and ultimate DISCO (uDISCO) (18). However, these protocols were still limited by fluorescence quenching of samples, incomplete clearing of specimens and not permitted antibody labelling, effectively. Attempts to address these issues and refine conditions of tissue processing have provided initial stimulation for optical clearing techniques.

A cutting-edge technique (termed CLARITY) developed by Chung and Deisseroth (19), provided a new tissue-processing platform for elucidating the 3D cellular connectome and arrangement in toto. This rapidly organ-clearing method, which has been the most common classical method for studying intact-tissue imaging and can be applied for probing molecular and structural underpinnings of intact tissue, largely broke through the limitations of using tissue-specific or application-specific reagents as described in prior clearing protocols. Since then, extensive research is accumulating on redesigning or optimization of clearing steps and reagents based on clarity method, including passive CLARITY (20), passive CLARITY technique (PACT) (21), active clearing technique- pressure related efficient and stable transfer of macromolecules into organs (ACT-PRESTO) (22), free-of-acrylamide and sodium dodecyl sulfate (SDS)based tissue clearing (FASTClear) (23), and fast freeof- acrylamide clearing tissue (FACT) (24).

Three clear contributions have been made via these techniques: stabilizing tissue structures using hydrogel embedding (19), use of fluorochrome signal- compatible clearing reagents (15) and large-scale and challenging tissue imaging improvement (25). Remarkably, of these approaches, application of the PACT and the FACT methods have been identified "effective" for their high-resolution and high-speed of clearing, simplicity, cost-effectiveness, until now (20, 24). Regarding the differences between the FACT and the PACT, the outstanding questions are: How does gel formation by acrylamide affect the preservation of protein of cell structure? What are the advantages and disadvantages of utilization of different concentrations of clearing solutions and different temperatures for solving acrylamide, for making tissues optically transparent? Another hallmark improvement in the FACTis removing polyacrylamide in its protocol; do the acrylamide and VA-044 initiator play redundant or parallel roles in the PACT, compared with paraformaldehyde used in the FACT? What are the negative effects of polyacrylamide? There are indeed some initial clues to address these questions regarding the characteristics of reconstructed 3D images of biological tissues in the FACT and the PACT protocols.

In this review, we summarize some important findings related to the FACT and the PACT for whole tissue imaging, with systematically comparing differences on recent published protocols between free-acrylamide method and PACT method for whole tissue imaging, including gel formation, clearing solution concentrations, clearing temperature, and negative effect of polyacrylamide. Before, it should be noted that this article discusses most of published research and review articles comparing or introducing acrylamide-based methods together or with other methods of clearing. It means the newly developed method, the FACT, has not been compared with acrylamide-based clearing methods. Based on comprehensive comparisons between the two tissue clearing techniques, FACT and PACT, we propose that FACT method performs better in the whole clearing processing, which may give novel insights into mapping the architecture of neural circuits and developing new approaches for tissue visualization. 

### Overview of two tissue clearing processes used for three-dimensional imaging

CLARITY technique has gained considerable success owing to its potential for mapping detailed structural and molecular information of intact biological systems and is supported by extensive research data, the applications of hydrogel embedding and electrophoretic tissue clearing (ETC) are at the very heart of the CLARITY clearing procedures associated with tissue preservation and clearing efficiency, both of these two techniques have had broad impact on the rate of tissue clearing, and had been incorporated into the design, or redesign, continually. 

Although active transport organ-electrophoresis approach which capitalizes on the highly charged nature of ionic micelles, can accelerate the lipid extraction by orders of magnitude, the payoffs of ETC are inevitable tissue degradation during sample heating and complexity to implement. Extending their prior work, Chung et al. (26) developed a novel approach (defined as passive CLARITY) (20) for intact tissues imaging without electrophoretic instrumentation, optimizing objectives and compatibility with light- sheet optics. Enormous studies have since done to understand the native biological molecules and fine structure underlying the passive CLARITY (Table 1).

Nevertheless, the main limitation of the slow rate of clearing makes the protocol impractical for scaling up, not to mention intact biological body mapping. On the basis of the passive CLARITY method, the concept of the PACT was first scientifically described by Yang et al. (21), when they presented a "passive tissue clearing and immunostaining" protocol for whole organisms with passive lipid extraction and proposed that such a method increased the speed of clearing, reduced tissue damage and promoted scalability. Furthermore, their work optimized reagents for the hydrogel embedding, clearing, and imaging, which provides important insights into how this technique is compatible with immunohistochemistry, endogenous-fluorescence. According to this modified passive CLARITY method, the PACT, corresponding successful applications have also been brought under the spotlight (Table 2). However, application of hydrogel caused tissue deformation in the clearing process, which results in adversely impacting the evaluation of fine cellular structures and long-term imaging.

**Table 1:** Successful applications of the passive CLARITY protocol for tissue clearing and three-dimensional imaging


Tissue/organ	Species	Hydrogel perfusion/embedding	Clearing solution	Clearing time	RI^*^ homogenization	References

Skeletal muscle (whole)	Mouse	+/+	4% SDS in boric acid (pH=8.5)	42 days (adult)	80% glycerol	(27)
Brain (whole)	Mouse	+/+	4% SDS in boric acid (pH=8.5)	21 days (adult)	FocusClear/85-87% glycerol	(20)
Brain (section)	Mouse	+/+	4% SDS in boric acid (pH=8.5)	7 days (adult)	PBST	(28)
Brain (whole)/lung (whole)/testis (whole)/kidney (whole)/intestine (whole)/spleen (whole)	Mouse	+/+	4% SDS in boric acid (pH=8.5)	30 days (adult)	FocusClear/80% glycerol	(29)
Brain (whole)/spinal cord (whole)	Mouse	+/+	4% SDS in boric acid (pH=8.5)	28-42 days (adult brain)/14-28 days (adult spinal cord)	TDE	(30)
Brain (whole)/spinal cord (whole)	Mouse	+/+	4% SDS in boric acid (pH=7.5)	36 days (adult brain)/21 days (adult spinal cord)	FocusClear	(31)
Brain (section)/spinal cord (section)	Mouse/rat	+/+	8% SDS in boric acid (pH=7.5)	4 days (adult mouse)/6 days (adult rat)	80% Glycerol/65% TDE	(32)
Brain (whole)	Rat	+/+	4% SDS in boric acid (pH=8.5)	28-56 days (adult)	RapiClear	(33)
Brain (section)	Rat	+/+	4% SDS in boric acid (pH=8.5)	6 days (age P0) to 20 days (age P24)	TDE	(34)
Brain (section)	Human	−/+	4% SDS in boric acid (pH=8.5)	14 days (adult)	ScaleA2 solution	(35)
Brain (whole)	Mouse/rat /human (section)	+/+	4% SDS in boric acid (pH=8.5)	21 days (adult mouse)/60 days (adult rat)/5-10 days (adult human)	87% glycerol/ScaleA2 solution	(36)
Cerebellum (whole)	Mouse/human (section)	−/+	4% SDS in boric acid (pH=8.5)	7 days (adult mouse)/>28 days (human adult)	RIMS+PBS+Tween-20	(37)
Spinal cord (whole)	Mouse	+/+	4% SDS in boric acid (pH=7.5)	14 days (adult)	CUBIC clearing solution	(38)
Whole body	Zebrafish	-/+	8% SDS in boric acid (pH=8.5)	5-7 days (adult)	RIMS	(39)
Fetus (whole)/brain (whole)/lung (whole)/heart (whole)/kidney (whole)/muscle^†^ (whole)	Mouse	+/+	4% SDS in boric acid (pH=8.5)	3-10 days (fetus)/10 days (other tissues)	RIMS	(40)
Liver (section)	Mouse	+/+	4% SDS in boric acid (pH=8.5)	30 days (adult)	RIMS	(41)
Retina	Rat	−/+	4% SDS	5 days	RIMS	(42)
Lung (whole)	Mouse	−/+	8% SDS in boric acid (pH=8.5)	ND	RIMS	(39)
Intestine (section)	Mouse/human	+/+	4% SDS in boric acid (pH=8.5)	12-14 days (adult)	80% glycerol	(43)
Ovary (whole)	Mouse	+/+	4% SDS in boric acid (pH=8.5)	35 days (adult)	FocusClear	(44, 45)
Testis (whole)	Zebrafish	−/+	8% SDS in boric acid (pH=8.5)	13 days (adult)	RIMS	(46)
Stem-cell-derived cortical cultures	Mouse	ND	ND	ND	ND	(47)


RI; Refractive index, SDS; Sodium dodecyl sulfate, TDE; 2,20-thiodiethanol, RIMS; Refractive index matching solution, PBS; Phosphate-buffered saline, ND; No data, PBST; Phosphate-buffered saline+Triton X-100, and †; The passive CLARITY was performed on muscle until the clearing stage and without immunolabelling and imaging.

**Table 2:** Application of successful PACT for different tissue clearing and three-dimensional imaging


Tissue/organ	Species	Hydrogel perfusion/embedding	Clearing solution	Clearing time	RI^*^homogenization	References

Brain (whole/section)/kidney (section)/heart (section)/lung (section)/intestine (section)/basal cell carcinoma (section)	Mouse/human	+/+	8% SDS in PBS (pH=7.5)	3-14 days (adult mouse brain)/ND (other tissues)	80% glycerol/RIMS/ sRIMS	(21)
Fetus (whole)/brain (whole)/lung (whole)/heart (whole)/kidney (whole)/muscle^†^ (whole)	Mouse	+/+	8% SDS in PBS (pH=7.5)	3-10 days (fetus)/25-30 days (other adult tissues)	RIMS	(40)
Brain (whole)/spinal cord (whole)/lung (whole)/heart (whole)/liver (whole)/stomach (whole)/salivary gland (whole)/pancreas (whole)/fetus (whole)/spleen (whole)/parotid gland (whole)/genital organ (whole)/kidney (whole)/bone (whole)	Mouse/Rat/Guinea Pig	+/+	8% SDS in PBS (pH=7.5)	17-23 days (adult)	nRIMS	(48)
Brain (section)	Mouse	-/+	8% SDS in PBS (pH=7.6)	1-3 days (adult)	RIMS	(49)
Intestine (section)	Mouse/ human	+/+	8% SDS in PBS (pH=7.5)	12-14 days (adult)	80% glycerol	(43)
Mammary gland (whole)/mammary tumor	Mouse	+/+	8% SDS in distilled water (pH=7.5)	4 days (adult)	sRIMS	(49)


RI; Refractive index, SDS; Sodium dodecyl sulfate, PBS; Phosphate buffer saline, PBST; Phosphate buffer saline+triton X100, RIMS; Refractive index matching solution, nRIMS; Nycodenz-based refractive index media solution, sRIMS; Sorbitol-based refractive index matching solution, TDE; 2,20-thiodiethanol, ND; No data, and †; The PACT is done on muscle until clearing stage and without immune-labeling and imaging.

**Fig.1:**
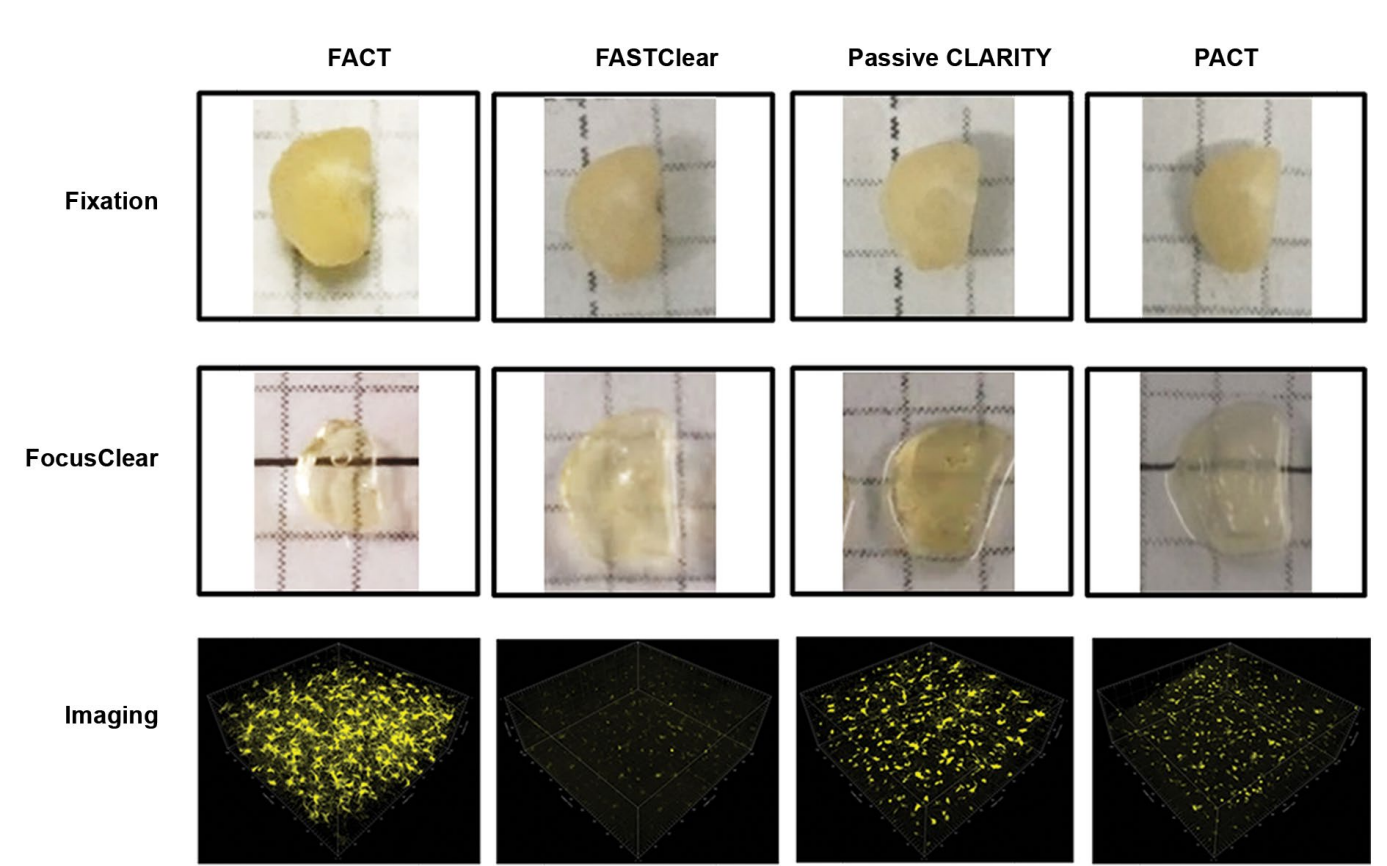
Whole-brain transparency comparison and representative fluorescence images of microglia obtained using different techniques. Images were adapted with permission (24).

Recently, an exciting new wave of improvements avoids incomplete tissue hydrogel hybridization and fine has emerged from free-of-acrylamide SDS-based cytostructure destruction in this protocol. However, the tissue clearing (FASTClear), which greatly reduces most prominent disadvantages of FASTClear is being the whole clearing time. Most notably, the replacement limited by immunolabelling to the full thickness of tissue. of acrylamide hydrogel by formaldehyde largely Moreover, problems of tissue shrinkage and archival formalin-fixed tissues clearing still exist. To challenge the above imperfections in 3D tissue imaging, simultaneously with FASTClear, the FACT (24) was introduced for tissue clearing and imaging pertaining to immunolabeled fluorescent or transgenic proteins by further merging and modifying the PACT (21) and the FASTClear (50) methods. In a compelling set of experiments, it has been demonstrated that the FACT further improves the speed of clearing, preservation of cytoarchitecture, depth of tissue penetration, long-term storage of fluorescent signal, the signal to noise ratio by comprehensively comparing the FACT protocol with the FASTClear methods, the PACT, and the passive CLARITY, demonstrating the higher potential of the FACT for rapid and high-resolution imaging on 3D biological tissue applications. A variety of successful applications of free-acrylamide tissue clearing are listed in Table 3. To better understand the optical transparency process of FACT, FASTClear and PACT, passive CLARITY, a more systematical comparison of workflow with these protocols has been shown in Figure 1.

**Table 3:** Application of successful free-acrylamide tissue clearing for different tissue clearing and three-dimensional imaging


Tissue/organ	Species	Hydrogel perfusion/embedding	Clearing solution	Clearing time	RI^*^ homogenization	References

Brain (section)	Mouse	-/-	8% SDS in PBS (pH=7.5)	3 days	FocusClear	(24)
Brain (section)	Mouse/human	-/-	4% SDS in sodium borate buffer	40 days(human)/ND (other tissues)	Histodenz-RIMS(mouse)47% 2,2’-Thidiethanol in 10 mM phosphate buffer (human)/ND (other tissues)	(52)
Brain (section)	Human	-/-	4% SDS in sodium borate buffer	Minimum of 5 days	FASTClear	(51)
Heart (section)	Dog/human	-/-	4% SDS buf fer	4 day	FASTClear	(23)


RI; Refractive index, SDS; Sodium dodecyl sulfate, PBS; Phosphate buffer saline, RIMS; Refractive index matching solution, and ND; No data.

### Role of gel formation by acrylamide and protein of cell structure 

Increase of our knowledge on gel formation that preserves proteins of cytoarchitecture but may conversely influence tissue expansion by clear solution, depth of antibody or light penetration in tissue, and speed of lipid removal is crucial. The hydrogel-embedding clearing protocols, including active CLARITY, passive CLARITY, PACT and so on, were developed based on polymerizing the fixed tissue into an acrylamide hydrogel prior to the process of lipid-removal. This hypothesis of hydrogel embedding provides a beautiful and simple account for how the use of hydrogel may provide physical framework for the cleared tissues and act as an impediment allowing for deep penetration of the antibodies into the tissue (30). It stems from one of the most influential reasoning that delipidation removes lipid bilayers which are essential for cellular integrity. Therefore, these formaldehyde- modified amines may crosslink proteins to acrylamide via nucleophilic addition reactions. Through this process, the acrylamide attached will be polymerized and form physical support to prevent excessive protein loss from samples in the steps of SDS delipidation, conversely, lipids and other biomolecules will be washed off due to the absence of the amine grouping (26). These features make hydrogel an extremely attractive crosslink substrate for maintaining cellular and molecular integrity of tissues thus they are vastly applied in researches on imaging of 3D tissues. Complications arisen in the past years, opened this hypothesis to debate (23, 24, 50, 51).

Interestingly, a more careful examination of different polyacrylamide concentrations in modified CLARITY protocol, found that the retention of RNA and penetration of antibodies improved with reducing acrylamide hydrogel composition from 4 to 1% (53). Furthermore, evidence began to mount that removing hydrogel from steps of perfusion and embedding can increase the amount, speed, and penetration depth of antibodies. Comparing the effects of samples morphologies between acrylamide-free and acrylamide-embedded tissues, which is consistent with the notion of reducing polyacrylamide concentration, revealed that using formaldehyde fixation singly could replace the acrylamide embedding of tissues for the steps of SDS-mediated delipidation and tissue clearing without affecting the tissue physical structure for high-resolution 3D histological analysis (52). Moreover, a series of problems using acrylamide hydrogel has also been brought under the spotlight. Tissues undergo expansion in the processing of polyacrylamide by SDS clearing and lose structural integrity as well (21, 36). Moreover, uncompleted hydrogel hybridization will block diffusion of hydrogel monomers when transcardial perfusion cannot be performed (17). On the basis of these observations, FASTClear protocol has been proposed for 3D visualization, and this technique has been successfully applied for human brain tissues (50) and myocardial tissue (23), so far. As reflected by a multitude of expounded theories, these modifications are SDS-based, avoid the use of acrylamide during gel formation, which are vastly simplified and more user-friendly, and range from overall processing time to immunostaining and visualization, leading to optimal tissue transparency conditions.

**Fig.2:**
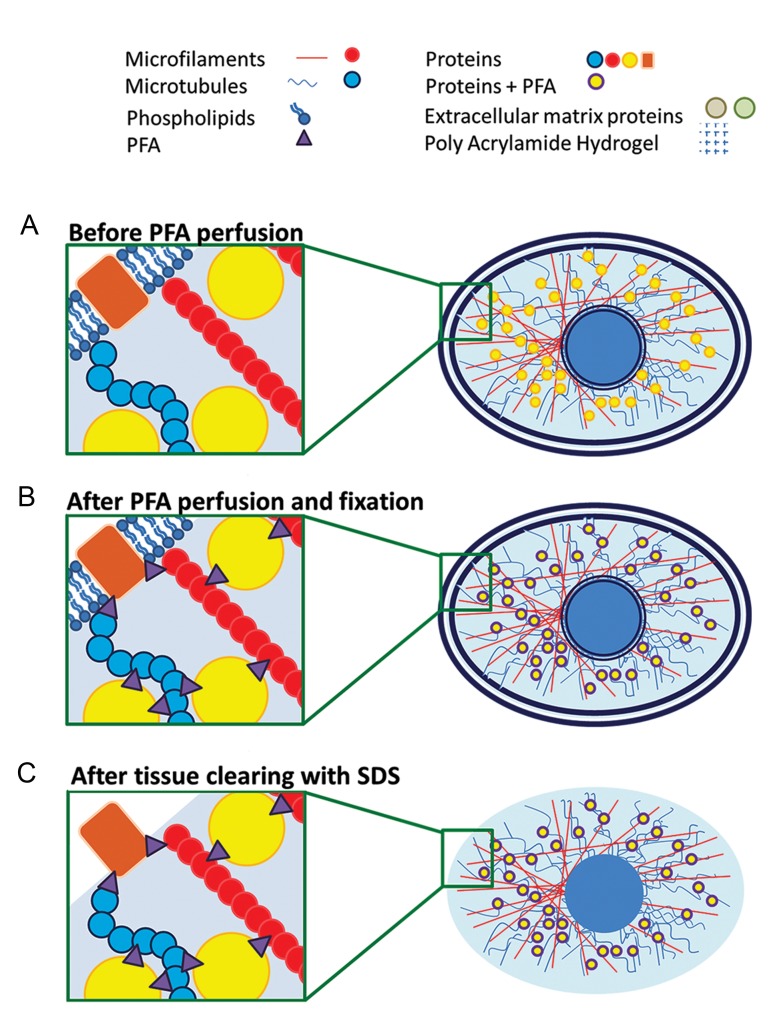
Mechanisms underlying the efficiency of the Fast Free-of-AcrylamideClearing Tissue (FACT) protocol. A, B. During the paraformaldehyde (PFA)- mediated tissue fixation in the FACT protocol, membrane and intracellularcytoplasmic proteins (including the transgenic fluorescent proteins) makechemical bonds with the cytoskeleton, including microfilaments and microtubules, and/or with the extracellular matrix, including proteoglycans. These bonds help to construct a massive 3D matrix that provides structuralsupport and essential tensile strength to the tissue during processing, and C. After removing the cell walls by 8% sodium dodecyl sulfate (SDS) in pH=7.5(optimum pH for preserving normal protein structures), the tissue scaffold ischemically bonded by PFA. Images were adapted with permission (24).

Simultaneous with the FASTClear, the FACT was developed (24) for further optimizing tissue clearing protocols for moving deep into fine cytoarchitectural details of brain cells in both genetically and chemically labelled tissues. Xu et al. (24) provided a systematic comparison between hydrogel-based and free-acrylamide clearing methods, which, for the first time, revealed that PFA-based FACT techniques can clear tissue faster. As it is shown in Figure 2, principles of PFA-based FACT methods may brought new insights into how different PFA serve to make strong chemical bonds with the cytoskeleton between proteins, which may challenge the classical theories that polyacrylamide serves to build cross-links between formaldehyde and hydrogel for assisting fixation of nucleic acids and protein upon the process of delipidation (54). 

**Fig.3:**
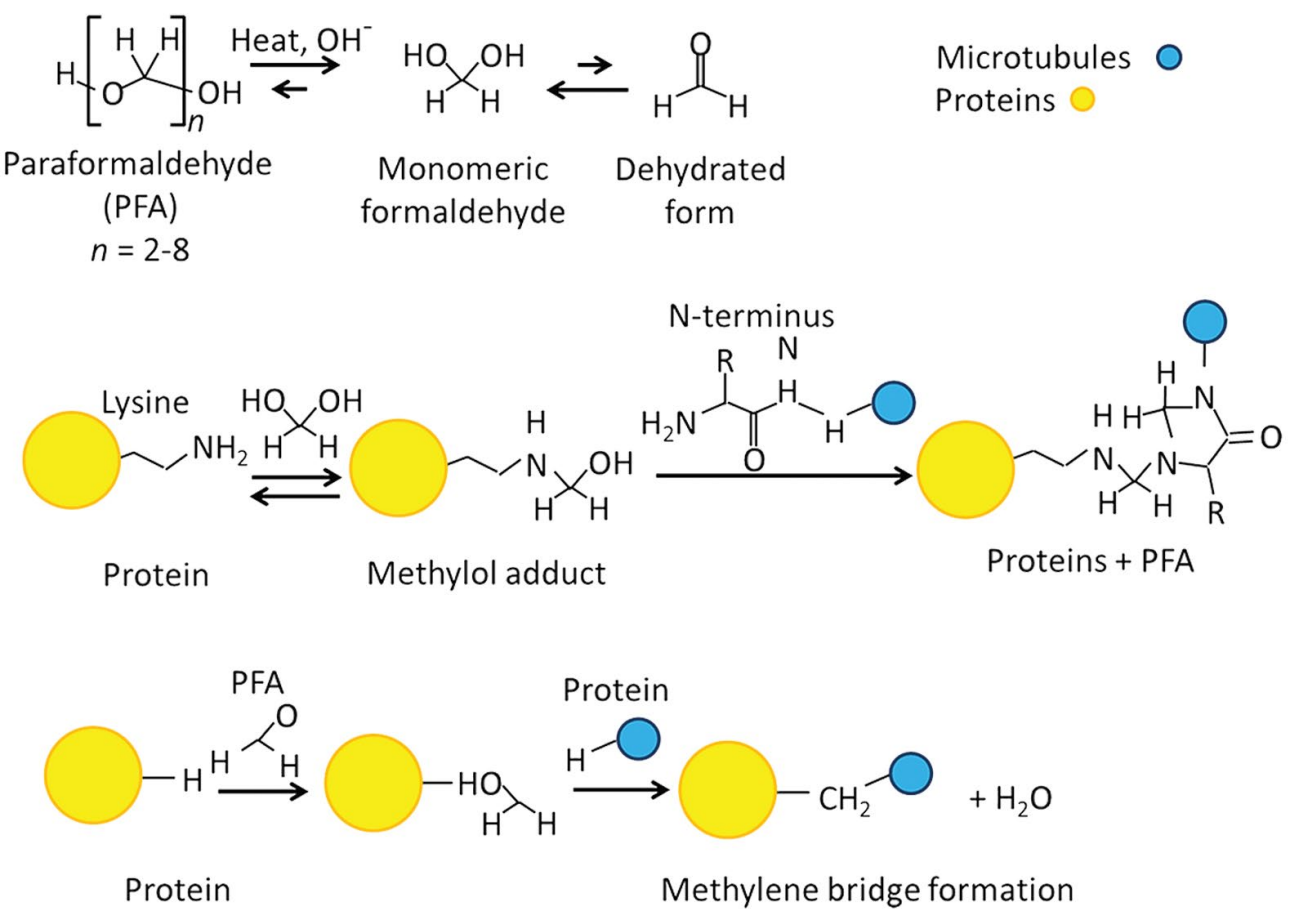
Chemical mechanisms through which paraformaldehyde crosslinks proteins. Images were adapted with permission (24).

One plausible explanation for PFA connection with peptides which is consistent with most of these data, is that aldehyde plays a crucial role in reaction with nitrogen or other atoms within proteins and peptides and therefore, creates a methylene bridge made of a -CH2- cross link (Fig .3). The majority of proteins are located in fixed places by these connections, which may form a large-scale 3D network. These kinds of bonds therefore provide essential tensile strength and structural rigidity of tissue upon harsh conditions. Most notably, although it has been reported that pores in polyacrylamide matrices motivate lipid exchange (21), no significant differences were observed in protein retention among these protocols. In a compelling set of experiments, a variety of related indicators, such as tissue weight, weight change ratio, the area change ratio were comprehensively measured in their experiments (24). There is considerable discrepancy between this discovery and prior works as significantly lower protein loss was observed in acrylamideembedded tissues upon the SDS delipidation step compared to non-hybridized tissues (26, 54). Expanding on this, given that not all proteins can be trapped in the extracellular matrix or intracellular space, such as cytoplasmic membrane or extracellular proteins, protein loss to some degree, cannot be avoided in all protocols, so far. Further experiments are needed to explore the potential possibility of having better protein preservation. Furthermore, based on our knowledge, there is no experiment defining the type of proteins removed during the clearing process.

### Different concentrations for different purposes (RNA and protein) 

The optimal methods for 3D tissue mapping depend on a set of processing factors that include selection of appropriate ingredients, optimization of pH, suitable incubation temperatures, thickness of sample, and perhaps also effort-based manipulation proficiency. For lipid removal, the clear solution composition at certain pH and solution concentration, could contribute to the speed of diffusion of detergent micelles and the rate of lipid solvation by detergent micelles in tissues (55). Each of several candidate detergents introduced during the past several years, either quenched native fluorescence (15, 55) or compromised tissue structure (9), opening these reagents to reexamination and debate (56). Useful operational comparisons were made by Yang et al. (21) in their PACT protocol. They tested various detergents at different concentrations for validity undergoing passively clearing brain tissue and revealed that SDS at all concentrations showed a superior effect for lipid solvation and delipidation as compared to other detergents. In traditional immunohistochemistry, SDS is proposed for protein denaturation for antigen retrieval. Delipidation properties of SDS have been also heavily implicated in tissue clearing in current years (20, 27, 34). Another intriguing discovery in their work is uniform clearing of the whole tissue only at 8% SDS concentration with less protein loss. Moreover, PACT studies also revealed that 8% SDS acts faster than 4% SDS in passive CLARITY technique. These features make SDS an extremely attractive reagent for tissue delipidation. Through this process, 8% SDS concentration is proposed for tissue clearing step in processing different samples for general applications. 

Early recordings revealed that the presence of free radicals which is associated with pH value of solution used in the tissue clearing process, closely affect the clearing speed (20, 21, 57-59), presumably reflecting that considerable heterogeneity of pH produces various effects during tissue clearing process. Some of these studies adopted the modifications of the clear solution with the inclusion of boric acid and a basic pH=8.5 for increasing clearing speed, which also increases protein loss at the same time. It can provide one reasonable explanation consistent with frequent observation of fluorescent bleaching in both hydrogel-based and PFA-based protocols, which used clearing solution of pH=8.5 in clearing step. However, by adjusting the clearing solution pH to 7.5, quenching of fluorescent transgenic labels was decreased (24), suggesting that pH=7.5 can optimize the effects of tissue clearing. Consistent with these correlative studies, further optimized preservation of fluorescent signal for tissue clearing was provided by adjusting pH at 7.5 and SDS solution concentration at 8% (24) specially for transgene labelled tissue.

### Different temperatures for removing polyacrylamide side effects 

Recent studies have also revealed that temperature is also one of the most sensitive factors during clearing incubation process to preserve the fluorescent signal. Exposure to high temperatures can accelerate delipidation of tissue; however, 50°C adopted in FASTClear method (50) was shown to induce yellowness or even burn or melt of the tissues reported in some protocols (20), resulting in increased tissue expansion, increased protein loss, and decreased imaging depth of endogenous florescent signals. In the FACT protocol (24), it was proven that a temperature of 37°C is crucial in non-acrylamide based protocol. Keeping pH at 7.5, temperature at 37°C and SDS concentration at 8% can reduce bleaching of transgene fluorescent signals and also remove lipids in a logic time period. All these modifications increased the quality of 3D tissue imaging in comparison with other methods (24). Temperatures >37°C in non-acrylamide-based protocols, despite increasing fluorescent signals bleaching, can increase tissue size and also increase protein loss such as observed in FAST Clear. 

### Negative effect of polyacrylamide on clearing (speed, protein loss, and tissue character)

The limitations of hydrogel crosslinking have substantial effects on the clearing process, including clearing speed, protein preservation, tissue character, which are thought to contribute to the index of clearing quality. The slow clearing speed using polyacrylamide has been brought under the spotlight. Theoretical basis of the FASTClear (50) postulates that the functions of polyacrylamide hydrogel in the intracellular and intercellular spaces, are not necessary to support sample spatial structure (19), but to impede clearing speed of removing of lipids by SDS. Moreover, tissue transparency techniques are much more diverse than originally thought, ranging from not only tissue with transcardial perfusion but also tissues that cannot be perfused. However, a large block of tissue could be limited by diffusion of hydrogel monomers which results in incomplete tissue hydrogel hybridization when samples are not suitable for transcardial perfusion (17). On the contrary, omission of polyacrylamide hydrogel from the process of tissue fixation facilitated lipid removal both in SDS 4% at 37% and the FACT protocols (24). Thus, as long as the tissue is well fixed in formaldehyde, free-of-acrylamide protocol is recommended to increase the speed of the tissue-clearing procedure.

Preservation of structural integrity is the most prominent factor of 3D tissue imaging, which is thought to provide an intuitive and detailed picture of how neural circuits and other connectomics work. Polyacrylamide was once considered to chemically incorporate native biomolecules into the hydrogel mesh, however, investigation of the necessity of hydrogel involvement has just started. Although, analysis based on daily measurement of tissue weight (served as an indicator of tissue and water absorption) and tissue size (served as an indicator of tissue size change) area revealed increased daily protein loss by removal of hydrogel from brain fixation comparing with the PACT and passive Clarity approaches. Intriguingly, the total protein losses showed no significant differences among hydrogel-based and FACT groups (24). This discrepancy could potentially be explained as follows: increased tissue clearing speed in FACT protocols decreased the days of protein loss and therefore caused minor lesion to protein preservation, comparing with hydrogel-embedded protocols with the lowest speed of tissue clearing.

Furthermore, the field has begun to appreciate that acrylamide-embedded tissues during SDS clearing undergo expansion and become more fragile because of loss of structural integrity (17, 36). Consistent with this hypothesis, extreme expansion and irreversible contraction of hydrogel are even more evident when absorbed water is removed from swelled tissue by FocusClear in hydrogel-embedded methods, inducing changes of cellular microstructures, such as microglia branches (17). However, the free-of-acrylamide protocols (24, 50) revealed that the omission of hydrogel from clearing procedure but relying on the structural support of cytoskeleton (lower water absorbance), can prevent deformities from polyacrylamide hydrogel expansion.

Therefore, PFA-based tissue clearing protocols can achieve a rapid clearing without influencing total protein loss and tissue area under specific conditions, comparing with hydrogel-based protocols. However, given that some degree of protein loss is inevitable in all protocols, and given the potential of more or less deformation of samples during lipid removing process occur no matter it is in PACT or FACT approaches. More effort should be made to further preserve more tissue structure details upon tissue transparency.

### Negative effects of polyacrylamide on staining (need specific antibody, non-specific staining, antibody penetration, and time consuming)

Avariety of unfavorable factors of polyacrylamide, such as specific antibody, non-specific staining, low antibody penetration, and being time consuming on staining, made it heavily implicated. According to hydrogel-based hypothesis, pores of polyacrylamide matrices assist lipid exchange and therefore enhance antibody penetration by changing hydrogel composition (21). This hypothesis has a major impact on providing conditions of antibody labelling of tissue, and application of transgenic labels in animal models. However, compared with protocols which are hybridized with hydrogel and PFA, it appeared that the immunolabelled tissues showed higher quality than transgene labelled ones (54). This discovery prompted the hypothesis of hydrogel crosslinking to proteins or network trapping of cellular proteins and therefore provided the possibility of trapping antibodies in immunohistochemical steps. Consistent with this hypothesis, background staining appear to increase after tissue washing, because antibodies are deeply trapped within tissue or they non- specifically bind primary or secondary antibodies. A possible explanation is that the low porosity of hydrogel contributes to non-specific accumulation of antibodies in dense tissues, which elicits non-specific labelling, as reflected by staining of striosomes in passive CLARITY technique (24, 60). On the contrary, absence of hydrogel in the FACT approach reduces these lesions during staining process. These observations are consistent with the discovery of FACT that the process of free-ofpolyacrylamide fixation and perfusion accelerate the immunostaining time of tissue and increase the depth of antibody penetration (24), which provide the possibility for better staining of full-thickness tissues.

### Negative effects of polyacrylamide on imaging (depth and light scattering)

Polyacrylamide hydrogel application in imaging are also subjected to modifications such as alteration of depth of penetration and light scattering, to increase background during imaging. Tissues are opaque for conventional light microscopy, mainly owing to their lipids, induce light scattering (15, 19) which occurs to materials with different refractive indices (RIs). The development of the tissue transparency technique stems from the theoretical principle that minimizing the light scattered by an object to achieve transparency. Lipids are the major source of light scattering in fixed brain, therefore, removing lipids or/and adjustment of the discrepancy of the RI between the surrounding areas and lipids, are potential approaches to increase sample transparency. Importantly, both transparency and integrity play a crucial role in achieving a high quality 3D imaging.

As mentioned above, hydrogel polymerization can be considered for the expansion and fragility of tissue during the imaging procedure. Deformation of tissue disrupts the evaluation of fine structures such as neuronal processes and microglia. In addition, lesions to tissue swelling is irreversible, a major adverse event for 3D tissue imaging quality, which RI cannot be corrected by using FocusClear. Abnormalities in tissue processing caused by polyacrylamide, heavily influence fluorescent signal intensity, antibody penetration depth as well as light scattering. In contrast, the absence of hydrogel in the FACT method circumvents these limitations and optimizes the preservation of antigenicity (24). Moreover, compared with other tissue clearing protocols (passive Clarity, PACT, and FASTClear), the FACT, which is based on optimizing all conditions in the whole procedure, is the most effective protocol in imaging procedure with the strongest signal detecting the depth of 300-700 µm. 

### Price, environmental concerns, and complications

Aided by merging and adjusting the FASTClear and PACT methods, Xu et al. (24) effectively simplified the tissueclearing protocol by removing acrylamide and VA-044initiator used during tissue clearing process and avoiding thesteps of degassing tubes with hydrogel solution, which hasprovided the most cost-effective and simplest protocol of 3Dtissue imaging with maximal preservation of fluorophoresignal, decreasing protein loss, and offering high speedclearing. Importantly, not using poisonous acrylamide for thepolymerization of hydrogel largely provided the researcherswith environment-friendly and less-toxic experiments. Furthermore, 3 to 5 times faster clearing of brain tissue by theFACT compared to PACT reduces SDS content in clearingsolution. Moreover, all reagents and solvents provided inFACT protocols are easily accessible in most laboratories. In addition, by deleting excess gel removing step in the FACT protocol in comparison with the PACT after gel formation, the fragile tissues such as fat tissue can be cleared and imaged by the FACT method. Being more accurate and simple, but less toxic, less laborious, and less time-consuming, contributed to the optimization of FACT protocol with respect to price, environmental concerns and complications.

### Concluding remarks and future perspectives 

We have witnessed tremendous progress in refining clearing methods introduced for identifying complex cytoarchitecture and mapping neural circuitry, involved in simplified protocols, higher quality and speed of data generation, safety and economical materials and reagents application. Notably, the PACT and the FACT methods have recently attracted considerable attention as they could optimize the clearing time, temperature, and reagents and preserve the fluorochrome signal. Notably, the FACT technique which has been developed by merging and modifying the FASTClear and PACT methods, includingavoiding hydrogel perfusion and embedding, decreasingthe temperature to 37°C, adjusting the clear solution pH to 7.5, has been identified with reinforcing properties compared with PACT for mapping detailed structures suchas neuronal processes and branches. As FACT facilitates thewhole workflow of tissue clearing including clearing timesaving, fluorescent signals preservation, cytoarchitectural retention, confocal microscopy optimization, data collection acceleration, the distribution of such a 3D tissue map provides a more intuitive brain tissue picture of how connections of large populations of cells and their networks and how physiological and pathological conditions lead to changes in expression of corresponding proteins and molecules. The FACT protocol suggests optimized conditions of rapid high- resolution imaging for brain tissue, which has made a big step over the PACTprotocol. In the following, we highlight several frontiers which should be addressed by further investigations to deepen our understanding of the FACT.

To test the validity and versatility of the FACT in 3D tissue imaging, a compelling set of experiments with tissue-type specificity and temporal precision recordings, remains warranted. Recently, a series of other tissue clearing methods has strongly established the visualization efficiency of various tissues including the spinal cord (61), whole embryo (62), bone (63), thymus, testis (22), pancreas, kidney, liver, intestine, lung (64), muscle (27), stomach, vasculature (65) and so on in different species including zebra fish (66, 39), mouse (10), rats (67), dogs (23), marmoset (15), and humans (21) in three dimensions. The FACT protocol has been tested on mouse, rat (68) and partridge (69) tissues for clearing various tissues of these species; nevertheless, reexamination of the proofs of necessity is still needed. 

Although some articles have been recently done using FACT protocol (68, 69); but it remains to be reexamined whether the FACT can be applied to general use other than brain tissue and whether tissue specificity affects tissue process and image quality. Furthermore, to specifically dissect the adaptability of the FACT protocol in large volume tissue imaging, it is necessary to resolve whether thick hard tissues -even whole body tissues- would abolish the advantages of the FACT in imaging quality of 3D tissue compared with other tissue clearing protocols. The FACT have been successfully applied for whole imaging, while only 1-mm-thick brain cortex slices handled with FACT method were reported for testing this property so far (24). The lack of such studies is perhaps owing to the difficulties in achieving a complete image of intact biological tissue due to the limitation of confocal microscope in a spatially and temporally precise manner. 

A potential problem of using scanning microscopes is that low scanning speeds make them impractical for imaging large fields of samples, resulting in light falloff, lens distortions, potential micro movements of the tissues inside the chamber (70). The molecular-structural evaluation of intact tissues therefore may not be precisely mapped using this kind of objective with high power but low working distance. Application of appropriate imaging setup, such as light sheet microscopy, can partly overcome this problem by enhancing optical penetration depth and accelerating data collection. Light-sheet microscopy, which selectively confines the illumination to the interest layer to achieve optical sectioning, are powerful, efficient and empirical methods used for reducing sample bleaching, large fields of view, high acquisition speed, and high dynamic range, compared with common classical method point-scanning microscopy (confocal or two- photon). For this, it will be helpful to image large volumes of tissue samples with the FACT approach.

## Conclusion

It would be an effective and reliable tool to simultaneously map subcellular molecular architecture between neighboring or distant cells of various organs, such as the brain and stomach, using multiple fluorophore signal in the same biological samples to examine how feedback loops and neuronal circuits works among central and peripheral system. Moreover, based on molecular- level and projection-based neural circuit tracing, it would be especially useful to comprehensively explore pathological and physiological conditions by comparing the functionality of protein and cell population. The advantages of the FACT protocol, such as maximal preservation of fluorophore signal hold promise for classifying and sub-classifying cytoarctitectures, and for molecular localization and projection in other projects.
